# Impact of MET targeting on tumor-associated angiogenesis and growth of MET mutations-driven models of liver cancer

**DOI:** 10.18632/genesandcancer.74

**Published:** 2015-07

**Authors:** Anne-Christine Piguet, Michaela Medová, Adrian Keogh, Astrid A. Glück, Daniel M. Aebersold, Jean-François Dufour, Yitzhak Zimmer

**Affiliations:** ^1^ Department of Hepatology, Department of Clinical Research, Inselspital, Bern University Hospital, and University of Bern, Switzerland; ^2^ Department of Radiation Oncology, Department of Clinical Research, Inselspital, Bern University Hospital, and University of Bern, Switzerland; ^3^ Department of Visceral Surgery, Department of Clinical Research, Inselspital, Bern University Hospital, and University of Bern, Switzerland

**Keywords:** MET, activating mutations, angiogenesis, small molecule inhibitors, liver cancer

## Abstract

Deregulated expression of the MET receptor tyrosine kinase has been reported in up to 50% of patients with hepatocellular carcinoma, the most abundant form of liver cancers, and is associated with decreased survival. Consequently, MET is considered as a molecular target in this malignancy, whose progression is highly dependent on extensive angiogenesis. Here we studied the impact of MET small molecule inhibitors on angiogenesis-associated parameters and growth of xenograft liver models consisting of cells expressing MET-mutated variants M1268T and Y1248H, which exhibit constitutive kinase activity. We demonstrate that MET mutations expression is associated with significantly increased production of vascular endothelial growth factor, which is blocked by MET targeting only in cells expressing the M1268T inhibitor-sensitive but not in the Y1248H inhibitor-resistant variant. Decrease in vascular endothelial growth factor production is also associated with reduction of tyrosine phopshorylation of the vascular endothelial growth factor receptor 2 expressed on primary liver sinusoidal endothelial cells and with inhibition of vessel formation. Furthermore, MET inhibition demonstrated an efficient anti-tumor activity and considerable reduction in microvessel density only against the M1268T-derived intrahepatic tumors. Collectively, our data support the role of targeting MET-associated angiogenesis as a major biological determinant for liver tumor growth control.

## INTRODUCTION

Liver cancers, and in particular the most prevalent form of these tumors, hepatocellular carcinoma (HCC), are aggressive, chemotherapy-resistant human malignancies, which exhibit an increasing annual incidence and are associated with poor prognosis [1, 2]. In that respect, liver tumors constitute the sixth most common human malignancy and the third most common cause of cancer-related mortality worldwide [3]. One of the most important reasons for the high mortality rate of patients with liver tumors is the absence of effective treatment options, especially in patients with advanced stages. Currently, the multi-kinase inhibitor sorafenib [4], which is believed to exert its major antitumor activity primarily by blocking the pro-angiogenic activity of vascular endothelial growth factor (VEGF) receptors 2 and 3 (VEGFR2 and VEGFR3), is the only approved treatment for advanced HCC and provides a median overall survival of 10.7 months in previously untreated patients [5-7]. Apart of primary tumors and due to being perfused by both arterial and venous blood, the liver often provides a unique microenvironment also to metastatic lesions of tumors of distinct histotypes [8].

Aberrant activation of the hepatocyte growth factor (HGF) receptor tyrosine kinase MET, mostly due to overexpression of the *MET* gene, is a common event in numerous types of human cancers and is frequently associated with poor prognosis [9]. In HCC, MET overexpression has been reported in 20-48% of tumor samples compared to peritumoral normal tissue [10-15] and was further correlated with clinicopathological features of these tumors such as presence of multiple nodular tumors and association with high proliferative index [10, 16, 17]. In addition, patients with MET overexpression usually have a significantly shorter 5-year survival than patients with low MET expression after a curative surgical resection [11, 17, 18].

On the basis of the critical role that MET signaling plays in human cancer, various small molecule tyrosine kinase inhibitors (TKIs) that block the tyrosine phosphorylation of the catalytic domain of the receptor with subsequent arrest of downstream signal propagation are currently under clinical trials [19, 20]. Most of the specific and non-specific MET TKIs function via competitively antagonizing occupancy of the intracellular ATP-binding site to prevent kinase domain phosphorylation. Few exceptions, such as ARQ197/tivantinib, bind to a region of MET outside of the ATP binding site and impair kinase activation through presumably conformational changes in a non-ATP competitive manner [21].

Apart of being directly correlated with definite tumor characteristics such as deregulated mitogenesis, motogenesis and resistance to apoptosis, various studies over recent years have highlighted the link between MET signaling and the formation of new blood vessels. An important work in this category by Zhang et al has substantially contributed to the understanding of the mechanisms that underlie MET-dependent tumor-associated angiogenesis by showing that MET signaling directly up-regulates transcriptional activation of the VEGF gene and in parallel down-regulates the expression of thrombospondin-1, an angiogenesis suppressing factor [22]. In the context of tumor progression for example, Garcia et al reported in 2007 that MET overexpression in breast carcinomas correlates not only with shorter survival, but also with tumor neoangiogenesis [23]. With respect to the impact of MET inhibition on tumor-associated vascularization, Puri et al were the first to show that growth attenuation of lung tumor xenografts in mice model by the MET small molecule TKI PHA665752 was associated with reduced blood vessels formation [24]. Similarly, Zou et al demonstrated that crizotinib, a non-specific MET TKI, which is currently in phase III clinical trial for the treatment of non-small cell lung cancer, was able to inhibit *in vitro* tubulogenesis of primary endothelial cells [25]. As potential interplay between aberrant MET signaling and angiogenesis in HCC, a study by Kaposi-Novak reported a MET aberrant expression-related transcriptional signature in a group of HCCs that were characterized by higher rate of vascular invasion and increased microvessel density [26], emphasizing therefore the potential role of MET-related angiogenesis in the pathogenesis of these tumors.

Taking into consideration the important emerging role of MET-dependent signaling in liver cancer pathogenesis with particular emphasis on MET-associated tumor vascularization, we investigated in the current study the potential of the MET TKIs to control growth and angiogenesis in models of MET-driven liver tumors, which consist of isogenic cell lines that ectopically express MET mutated variants that differ exclusively by their responsiveness to MET targeting.

## RESULTS

### M1268T and Y1248H MET-mutated variants display different sensitivities towards PHA665752

**Figure 1:**
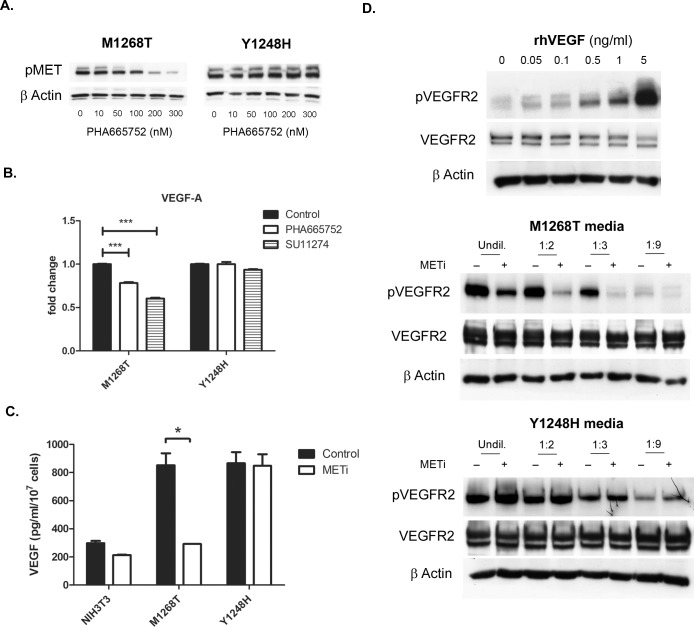
Impact of MET inhibition on VEGF signaling **A.** Effect of PHA665752 treatment (0-300nM for 16 hrs) on phosphorylation (Tyr1234/1235) of MET in NIH3T3 cells expressing the MET M1268T (left) and the MET Y1248H (right) mutation. **B.** VEGF mRNA levels in NIH3T3 MET M1268T and Y1248H following MET targeting by PHA665752 (50nM for 24 hrs) or SU11274 (2μM for 24 hrs) (***, p<0.0005). **C.** Effect of MET inhibition (METi; 50nM PHA665752 for 24 hrs) on MET-dependent production of VEGF (*, p<0.05). **D.** Impact of rhVEGF (upper panel), conditioned media collected from NIH3T3 MET M1268T cells (middle panel) and conditioned media collected from NIH3T3 MET Y1248H cells (lower panel) on VEGFR2 phosphorylation in LSECs (METi – PHA665752-treated cells, Undil. – undiluted medium, 1:2, 1:3, 1:9 – 2, 3 or 9 times diluted conditioned media).

The prime interest of the current study was to evaluate *in vitro* as well as in an orthotopic liver tumor model the potential of two MET small molecules inhibitors, SU11274 and PHA665752 to interfere with tumor-associated angiogenesis. To that end we used isogenic NIH3T3 mouse fibroblast cells expressing the activating MET-mutated variants M1268T and Y1248H, which display *in vitro* sensitivity and resistance respectively towards SU11274 [27]. As suggested in Figure 1A (left panel), cells with the M1268T activating point mutation showed a dose-dependent decrease in MET phosphorylation upon PHA665752-treatment, similarly to the previously described effect of SU11274 [27]. In contrary, no change in MET phosphorylated status was observed in Y1248H cells treated with increased concentrations of PHA665752 (Figure 1A, right panel). This indicates analogous inhibitory potential of SU11274 and PHA665752.

### Impact of MET inhibition on production of VEGF

In the last years, it has been reported that one of the biologic activities exerted by HGF on tumor cells is up-regulating the expression of the pro-angiogenic factor VEGF [28, 29]. In this section, we aimed to investigate whether the same is also valid for constitutively active oncogenic variants of MET and whether blocking of MET interferes with the production of VEGF by these cells. Initially, the impact of MET inhibition on VEGF mRNA levels has been investigated in NIH3T3 MET M1268T and Y1248H cells via RT-PCR analysis. As Figure 1B indicates, both PHA665752 and SU11274 significantly reduced VEGF mRNA levels only in the M1268T variant cells with no apparent impact on cells expressing the Y1248H MET inhibition-resistant variant. We further incubated parental as well as NIH3T3 cell lines expressing MET M1268T and Y1248H activating mutations in presence or absence of the MET inhibitors SU11274 and PHA665752 for 24 hours. The levels of VEGF that were secreted by the cells were then measured in collected medium by ELISA. To exclude an effect due to different cell numbers caused by growth arrest impact of the MET inhibitors, the cells were counted when the media was collected and the VEGF amount was normalized to the cell counts.

Interestingly, we found that ectopic expression of MET in NIH3T3 was accompanied by a considerable increase in the production of VEGF of approximately 3-fold compared to parental cells, confirming the previous observations of MET-dependent VEGF transcriptional upregulation [22]. Furthermore, while the MET inhibitor PHA665752 had no effect either on the production of VEGF in the drug-resistant cells expressing MET Y1248H variant or in the parental NIH3T3 cell line, the production of VEGF was significantly reduced upon PHA665752 as well as SU11274 treatments in the cells carrying the inhibitor-sensitive MET mutation M1268T (Figure 1C and data not shown). In this case, the inhibition of MET-dependent transcription of VEGF led to a significant, 2,9-fold reduction of VEGF levels in culture medium (p<0.05).

### In vitro pro-angiogenic effects of conditioned media from MET-driven tumor cells are diminished upon MET inhibition

We further aimed to translate the findings from the previous section and to investigate the impact of the MET-dependent VEGF levels modification on biological endpoints. We tested the potential of conditioned media from PHA665752-treated and untreated M1268T and Y1248H cells to induce the phosphorylation of VEGF receptor type 2 (VEGFR2) on the surface of endothelial cells by Western Blot. Liver sinusoidal endothelial cells (LSECs) were stimulated with various dilutions of VEGF-containing conditioned media. As positive control for this system, the impact of human recombinant VEGF (rhVEGF) on VEGFR2 phosphorylation in LSECs was tested (Figure 1D, upper panel). As shown in Figure 1D, middle panel, we observed a remarkable decrease in VEGFR2 phosphorylation in the cells stimulated by media collected from PHA665752-treated, inhibitor-sensitive MET mutants compared to media from control cells. On contrary, there was no difference in VEGFR2 phosphorylation status of LSECs using media from treated and untreated Y1248H cells (Figure 1D, lower panel).

**Figure 2:**
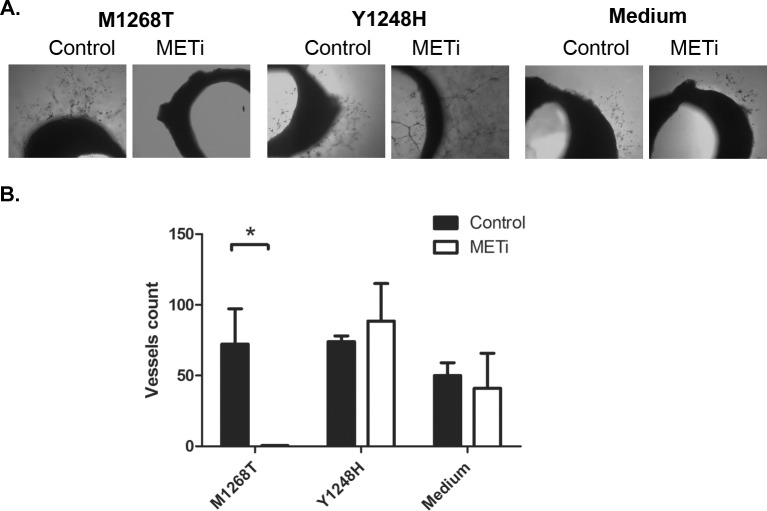
In vitro angiogenesis studied by the rat aortic ring assay **A.** Representative pictures of rings incubated with conditioned medium from NIH3T3 MET M1268T (left), NIH3T3 MET Y1248H (middle) cells or medium only (right) incubated with MET inhibitor (METi; SU11274). **B.** Vessels quantification. The vessel number was reduced after addition of medium from NIH3T3 MET M1268T cells treated with SU11274 (2μM) compared to medium from untreated cells (N=3; *, p<0.05). Exposure to medium obtained from the MET-inhibitor treated NIH3T3 MET Y1248H cells slightly increased the number of vessels compared to medium from untreated cells.

Further, the angiogenic effect of the culture media from treated or untreated NIH3T3 M1268T and Y1248H cells was assessed using the rat aortic ring assay. Dissected aortas of Wistar rats were placed onto matrigel which allows the sprouting of capillaries. As shown in Figure 2, the sprouting of vessels was completely abolished when rat aortic rings were incubated with medium coming from M1268T cells treated with MET inhibitors, compared to incubation with the medium of untreated M1268T cells. In contrast, this effect was not observed with media obtained from treated, inhibitor-resistant Y1248H cells.

### PHA665752-mediated reduction of the size and the associated microvessel density of MET M1268T tumors in an orthotopic liver tumor model

To investigate whether the effects observed *in vitro* are of *in vivo* significance, NIH3T3 MET M1268T or Y1248H cells were injected subcapsularly into the liver of SCID mice. One week after injection, mice were randomized for PHA665752 (25mg/kg/day i.p.) or vehicle treatment. After seven days of treatment, tissues were harvested and tumor sizes and weights measured.

**Figure 3:**
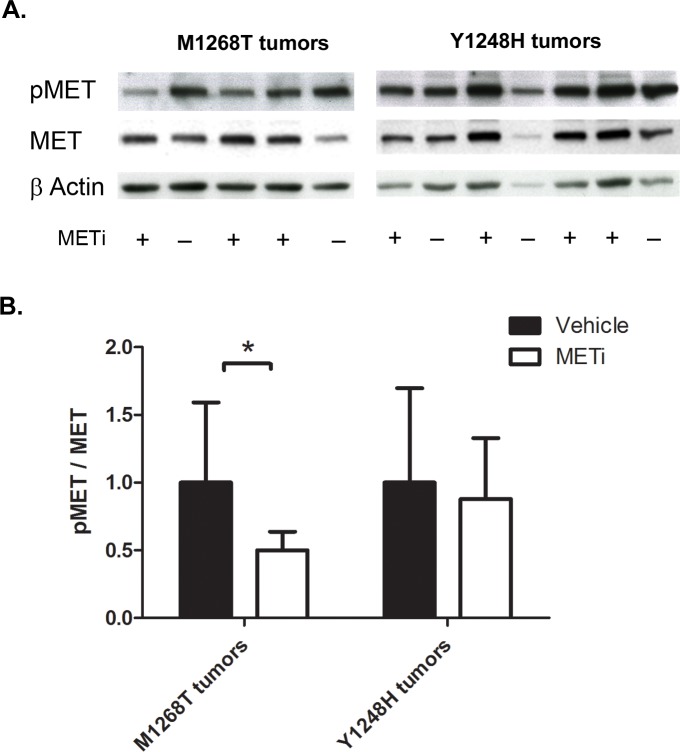
Effect of METi on the phosphorylation status of NIH3T3 MET M1268T and Y1248H liver tumors **A.** Western blot showing the phosphorylation status and the expression levels of MET in control and in PHA665752-treated (METi) NIH3T3 MET M1268T (left) and Y1248H (right) liver tumors. −, vehicle-treated liver tumors; +, PHA665752-treated (METi) liver tumors. **B.** Quantification of pMET/MET expression normalized to β actin (N=5; *, p<0.05).

As aforementioned, PHA665752 acts by inhibition of the MET receptor tyrosine kinase activity and subsequently of its downstream signaling. To confirm that the effects observed on tumor xenografts were indeed mediated by PHA665752, we studied the phosphorylation status of MET in harvested tumors by Western blot analysis. In M1268T tumors, the phosphorylation of MET was significantly decreased (down to 50% of the non-treated samples, p<0.05) when the animals were receiving PHA665752 compared to vehicle-treated mice. By contrast, the treatment with PHA665752 reduced the phosphorylation of MET in Y1248H tumors only by 10% (Figure 3, p>0.05).

**Figure 4:**
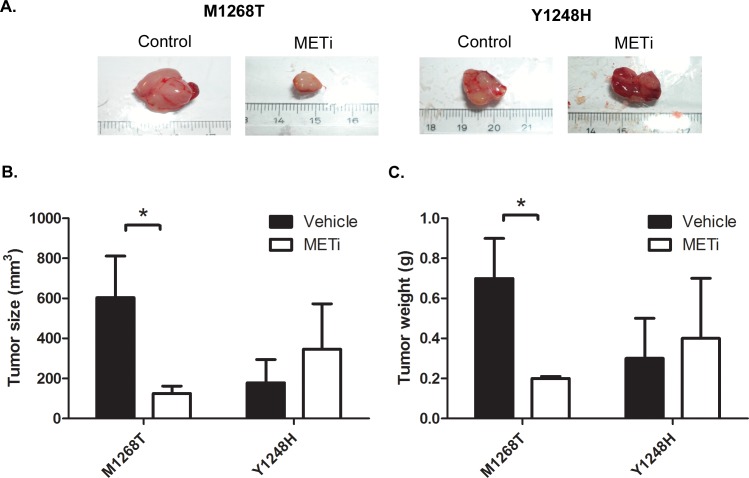
Impact of METi treatment on the size and weight of liver tumors formed by NIH3T3 MET M1268T and Y1248H cells **A**. Representative pictures of vehicle-(CONTROL) or PHA665752-treated (METi; 25 mg/kg/day) NIH3T3 MET M1268T (left) and Y1248H (right) liver tumor **B.** Quantification of tumor sizes (N=3; *, p<0.05), **C.** Quantification of tumor weights (N=3; p<0.05).

The size of PHA665752-sensitive M1268T tumors was significantly reduced in the animals treated with the MET inhibitor, compared to vehicle-treated mice (Figure 4A and 4B). In contrast, the treatment with PHA665752 had no effect on the size of the Y1248H liver tumors (Figure 4A and 4B). Specifically, M1268T tumors measured 604±207 mm^3^ in untreated mice and 125±37 mm^3^ in PHA665752-treated mice (p<0.05) while Y1248H tumors measured respectively 178±116 mm^3^ and 346±227 mm^3^ (p>0.05). Comparing tumor weights, very similar effect of reduction upon PHA665752 treatment was observed in the case of M1268T sensitive liver tumors (0.7±0.2g in untreated animals vs. 0.2±0.01g in animals treated by the MET inhibitor (p<0.05); Figure 4C). Similarly to tumor size, there was a slight but not significant increase in the weight of Y1248H tumors upon PHA335752 treatment (0.3±0.2g vs. 0.4±0.3g; Figure 4C).

**Figure 5:**
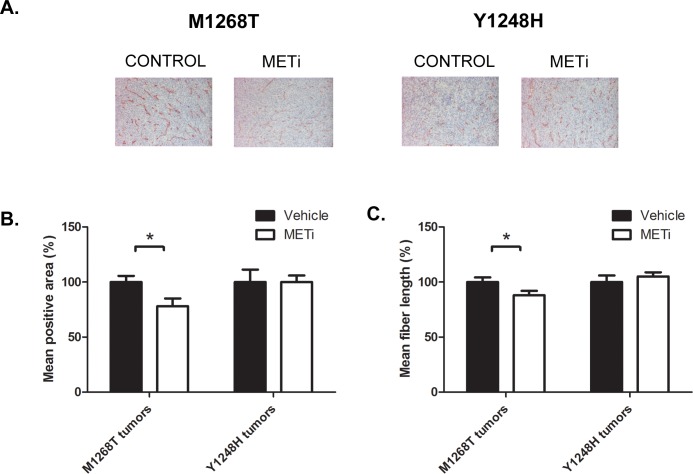
Impact of METi on the microvessel density in NIH3T3 MET M1268T and Y1248H liver tumor sections **A.** Representative pictures of CD31 immunostaining of vehicle-(CONTROL) or PHA665752-treated (METi; 25 mg/kg/day) NIH3T3 MET M1268T (left) and Y1248H (right) liver tumor sections. **B.** Quantification of CD31 mean area of positive signal per section (N=7; *, p<0.05), **C.** Quantification of mean fiber length (N=7; *p<0.05).

To assess the impact of MET inhibition on tumor angiogenesis, we evaluated the state of blood vessels in the tumor sections by immunohistochemical staining using an antibody against the endothelial cell surface marker CD31 (Figure 5A). Differences in CD31 staining upon MET inhibition were evaluated by assessing the mean area of CD31 positive zone and the mean vessels fiber length. The results shown in Figure 5B demonstrate that the mean area of CD31 positive signal was significantly reduced by 22% following PHA665752 administration in the MET inhibition-sensitive M1268T tumors whereas the drug had no corresponding effect in the resistant Y1248H tumors. A statistically significant reduction of 12% in the mean of vessels fibers length was also observed in the drug-sensitive M1268T tumors (p<0.05), with no parallel decrease in Y1248H-associated tumors (Figure 5C).

## DISCUSSION

Liver cancers and in particular HCCs are hypervascularized tumors [30], a feature that highlights their particular potential sensitivity towards antiangiogenic targeting treatments. Indeed, this biologic rationale along with improvement of survival of patients with advanced HCC [6, 7] underlies the clinical application of the angiogenesis inhibitor sorafenib for the treatment of these malignant disorders. However, despite initial responses to sorafenib, potential escape mechanisms are probably responsible for disease relapses in most of the patients [31]. Another evidence for the benefit for interference with tumor-associated angiogenesis in liver cancers is provided by data from clinical and pre-clinical studies with mTOR inhibitors such as everolimus and sirolimus. mTOR inhibitors are believed to exert their antiangiogenic effects largely through regulation of VEGF expression and translation of other proteins involved in angiogenesis [32, 33]. In that respect, administration of everolimus in patients with advanced HCC has led to a median progress-free survival of 3.8 months with a disease control rate of 44% in phase I/II tests [34]. Moreover, administration of the anti-VEGF receptor 2 antibody ramucirumab in second line after sorafenib demonstrated a benefit in a subgroup of patients with HCC. Nevertheless, the current data that emerge from experiences with sorafenib strongly justify the identification of additional targeting approaches that exhibit among other effects also antivascularization properties, for the handling of liver malignancies.

In agreement with the role that aberrant biologic activity of the MET/HGF axis may play a role in the pathogenesis of liver tumors, there is a concomitant increase in the interest in MET as a molecular target in these malignancies [35]. Indeed, emerging pre-clinical data support the potential of MET as a clinical target in liver tumors. For example, it was recently described by Inagaki et al that the prototypic MET TKI SU11274, which was used also in the current study, suppressed HCC cell growth by inhibiting the activation of the receptor [36]. In this study, it was demonstrated that SU11274 suppressed the growth of HepG2, HuH7, and HLE hepatoma cells with IC_50_ values of 6–8 μmol/L *in vitro*. Expression of phosphorylated MET and of MET-dependent phosphorylated ERK gradually decreased in a dose-dependent manner with SU11274. Concerning clinical evaluations, the most advanced compound in this category at present is tivantinib/ARQ197, an oral MET small molecule, which was identified as the first non-ATP competitor MET TKI [21]. Even though the exact mechanisms by which tivantinib exerts its activity have been questioned recently [37, 38], suggesting that in addition to MET inhibition, this TKI exhibits also a tubulin-associated cytotoxic activity, other studies indeed furnished data confirming the tivantinib anti-MET targeting capacity [39-41]. Importantly, matched tumor samples from patients before and after treatment with tivantinib demonstrate MET inhibition [42]. Recent results of a phase II study suggest that tivantinib may be beneficial for patients with MET-positive HCC who have failed or show sorafenib-intolerance [43]. Another MET TKI that is evaluated in preclinical models of HCC is XL-880/foretinib [44].

Despite the role of MET signaling in tumor angiogenesis [24, 25, 45] and the possible therapeutic benefit of targeting MET aberrant expression in liver tumors, the impact of MET inhibition on liver cancer-associated angiogenesis has not been yet adequately reported. Consequently, in the current study we aimed to investigate the impact of aberrant MET activity targeting on pro-angiogenic activities in MET-driven tumor cells and in a liver xenograft model. In order to attribute directly the MET-dependent pro-angiogenic activities and in order to exclude off-target activities, we have chosen to use NIH3T3 cells that ectopically express constitutive active MET variants, which display sensitivity or resistance towards the MET TKIs SU11274 and PHA665752. The first interesting finding in the current report is that oncogenic MET activity is sufficient to induce production of VEGF, as indicated by the nearly 3-fold increase in VEGF in conditioned media of cells expressing the MET mutated variants as compared to parental cells. This finding matches well with the original study by Zhang et al. who were the first to demonstrate an HGF-dependent increase in VEGF expression in leiomyosarcoma and breast cancer cell lines [22]. Our data concerning the impact of MET inhibition on reduction of VEGF production by the MET M1268T cells are in accordance with the study by Puri et al who found a considerable reduction in VEGF expression in lung cancer xenografts in mice following treatment with the MET TKI PHA665752 [24]. Similar results were shown by Zou et al who reported reduction of the pro-angiogenic factors VEGF and IL-8 in response to the MET small molecule PF-02341066 in tumor models derived from GTL-16 and U-87 cell [25]. *In vivo*, we could demonstrate that MET inhibition was bound to a decrease in the size and in the vascularization of tumor in a murine liver xenograft model. Altogether, the data of this manuscript provide further support for the importance of blocking of MET-associated angiogenesis in a hypervascularized microenvironment as that of the liver for efficient tumor growth control.

## MATERIALS AND METHODS

### Cell culture

Parental NIH3T3 cells as well as NIH3T3 cell lines, which stably express the MET-mutated variants M1268T and Y1248H were obtained from Dr. Laura Schmidt (National Cancer Institute NCI, Fredrick, MD, USA) and maintained in DMEM (GIBCO, Invitrogen Corp.) supplemented with 10% FCS (Sigma), antibiotic-antimycotic (penicillin 100U/ml, streptomycin sulfate 100U/ml, amphotericin B as Fungizone 0.25ug/ml GIBCO, Invitrogen Corp.). In addition, medium for MET-mutated cells contained 0,5mg/ml Geneticin/G-418 sulfate (GIBCO, Invitrogen Corp.).

Primary liver sinusoidal endothelial cells (LSECs) were kindly provided by the laboratory of Dr. Deborah Stroka (Department of Clinical Research, University of Berne, Switzerland) and were cultured on plastic coated with collagen in Human Endothelial-SFM Basal Growth Medium (GIBCO, Invitrogen Corp.) supplemented with GPS, 20% Human Serum, HGF (final concentration (f.c.) of 10ng/ml) and VEGF (f.c. 2ng/ml).

### Small molecules

Small molecules SU11274 [(3Z)-N-(3-chlorophenyl)-3-({3,5-dimethyl-4-[(4-methylpiperazin-1-yl)carbonyl]-1H pyrrol-2-yl}methylene)-Nmethyl-2-oxoindoline-5-sulfonamide] as well as PHA665752 (3*Z*)-5-[(2,6-dichlorobenzyl)sulfonyl]-3-[(3,5-dimethyl-4-([(2*R*)-2-(pyrrolidin-1-ylmethyl)pyrrolidin-1-yl]carbonyl)-1*H*-pyrrol-2-yl)methylene]-1,3-dihydro-2*H*-indol-2-one were obtained from SUGEN, Inc. ((Pfizer) South San Francisco, CA, USA).

Stock dilutions of the drugs (10mM) were prepared in dimethylsulfoxide (DMSO) and stored in solution at −20°C. Final dilutions for cellular assays were prepared immediately before use in the corresponding media.

### Antibodies

Rabbit polyclonal anti-phospho-MET (Tyr1234/1235) and rabbit monoclonal anti-phospho-VEGFR2 (Tyr1175) and anti-VEGFR2 antibodies were obtained from Cell Signaling (Cell Signaling Technology, Inc., Beverly, MA, USA). Rabbit polyclonal anti-MET (SP260) antibody was purchased from Santa Cruz Biotechnology, Inc., Santa Cruz, CA, USA. Rabbit polyclonal anti-beta-actin antibody was obtained from Sigma (Fluka, Buchs, Switzerland). Rat monoclonal anti-mouse CD31 (clone JC/70A) antibody was purchased from Dako, Glostrup, Denmark.

### Conditioned media

NIH3T3 cells expressing the MET-mutated variants M1268T and Y1248H were plated and incubated in the presence or absence of MET small molecule inhibitors PHA665752 (100 and 200nM) and SU11274 (2μM). Twenty-four hours later, the conditioned media of the cells were collected, centrifuged and used for further experiments.

### Determination of VEGF concentration in conditioned media

The concentration of VEGF in the culture media was measured according to the instructions of the ELISA Mouse VEGF kit (Mouse VEGF Quantikine, R&D Systems Europe, Ltd., Abingdon, UK) in flat bottom transparent polystyrol microtiterplates. The reaction was stopped with 2N H_2_SO_4_ and hydrogen peroxide in a buffered solution together with 3,3′,5,5′ tetramethylbenzidine in organic solvent (BD PharMingen, Heidelberg, Germany) were used as substrate solutions. The absorbance at 450 nm (with 570 nm correction) was read using Infinite^TM^ 200 Tecan (Tecan Group Ltd.) and the concentration of VEGF was determined according to standard curves. The amount of VEGF produced by the cells was normalized to the count of cells following the collection of the media.

### RNA isolation and Real-time PCR

Total RNA was extracted from cultured cells using Trizol reagent following the manufacturer's instructions (Roche). Reverse transcription of mRNA was performed using the Omniscript RT Kit (Qiagen). Quantitative PCR of VEGF-A was assessed using a 7900HT fast Real-time PCR system (Applied Biosystems) using a TaqMan assay (Applied Biosystems). The mean C_T_ was determined from triplicate experiments, mRNA levels were normalized to the level obtained for TATA-box-binding protein (TBP). Changes in expression were determined by calculations of ßßC_T_.

### Cell stimulation and immunoblot analysis

After reaching approximately 60% confluency, LSECs were incubated in starvation medium (0.1% HCS, without growth factors) for 16 hours and then stimulated for 10 minutes at 37°C with various dilutions of conditioned media obtained from treated as well as untreated NIH3T3 MET-mutated cells. LSECs were then lysed by sonication and lysates were loaded together with SDS loading buffer (Bio-Rad Laboratories, Inc.) onto 7% SDS-polyacrylamide gel electrophoresis.

NIH3T3 MET-mutated cells were treated for 16 hours with various concentrations of PHA665752 and lysed in lysis buffer (1% Triton X-100, 0.5% Nonident NP-40, 1mM EGTA, 1mM EDTA, 150mM NaCl, 10mM Tris-HCl, 1mM Na_3_VO_4_, 10mM NaF, 1mM ZnCl_2_, 50mM Na_2_MoO_4_, 1.4mg/ml aprotinin, 35ng/ml PMSF, a cocktail of proteases inhibitor (Complete Mini, Roche)).

Tissues were homogenized in 0.25 M sucrose (Polytron; Janke&Kunken KG IGA Werk, Staufen, Germany).

Protein concentration was determined by using the BioRad protein quantification reagent (Bio-Rad Laboratories, Inc.). Fifty micrograms of total protein were resolved by SDS-PAGE on a 7% gel and transferred onto a PVDF membrane. After blocking and incubating with specific primary antibodies, membranes were incubated with horseradish peroxidase-linked secondary antibody (Amersham Pharmacia Biotech, Piscataway, NJ). Detection of secondary antibodies was performed using the ECL kit (Amersham) and autoradiography.

### Aortic ring assay

Thoracic aortas excised from 8- to 12-week-old ACI rats (range 180-230 g) were cut into 1-mm-long cross-sections. Rings were placed on matrigel-coated wells with F12-K medium (Gibco, Basel, Switzerland) with 10% fetal bovine serum (Sigma-Aldrich Chemie GmBH, Munich, Germany), penicillin, streptomycin and supplemented with 10ng/ml epidermal growth factor and 25μg/ml heparin. Conditioned media from SU11274-treated as well as untreated NIH3T3 cells were added. After 5 days, rings were fixed and stained using a Diff-Quick solution II staining protocol (Diff-Quick Stain Set; Baxter-Dade AG Düdingen, Switzerland). Vascular outgrowth was quantified by counting all sprouts from one ring. All assays were performed in triplicate and each experiment was repeated three times.

### In vivo studies

NIH3T3 MET M1268T or Y1248H cells (2,5×105 cells/animal) were injected subcapsularly into the liver of 12-18 weeks old SCID mice (Harlan, Indianapolis, USA). One week after implantation, mice were randomized for PHA665752 (25mg/kg/day i.p.) or vehicle treatment. After seven days of treatment, tissues were harvested and tumor sizes and weights measured. Animals received humane care in accordance with the regulations for laboratory animals and the experiments were performed following protocols approved by the animal use committee of the Canton of Berne, Switzerland.

### Microvessel density

The vascularization of tumors was quantified by CD31 immunohistochemistry. The microvessel density (mean area and mean fiber length) was determined blindly as described by Weidner and Folkman [46].

### Statistics

If not stated differently, statistics results are presented as mean +/− standard deviation. Comparisons were performed with a non-parametric Mann-Whitney test and Student T test.
